# Pearls and paradigms in Infective Keratitis

**Published:** 2019

**Authors:** Manisha Acharya, Javed Hussaina Farooqui, Shikha Jain, Umang Mathur

**Affiliations:** * Dr. Shroff’s Charity Eye Hospital, New Delhi, India; ** Ram Manohar Lohia Hospital, New Delhi, India

**Keywords:** infective keratitis, bacterial, management, microbiology, staining, culture, treatment

## Abstract

Infective Keratitis is a commonly encountered sight-threatening ocular emergency. In developing countries, it is a major cause of corneal blindness. Empirical treatment without microbiological work up often leads to treatment failure. Indiscriminate use of steroid antibiotic combination worsens the situation and makes further management challenging. The correct line of management can be potentially sight saving for both the ophthalmologist and the patient. This article on Infective keratitis has been written keeping best practices and protocols in mind. In a very simple and concise form, it focuses on the salient features of clinical presentation of infective keratitis and the stepwise approach to subsequent management in a patient. It explains in detail the way to perform corneal scraping, the importance of the same and further management based on microbiologically proven result. The management part includes indications and methods for medical as well as surgical intervention. We aimed to share our experience in the management of patients presenting with infective keratitis in the clinic.

## Natural Ocular Defenses

The eye is protected from all sides by the projecting bony orbital rim, and additionally by the eyelids [**1**]. A healthy tear film with a normal lacrimal drainage system serves as another defense, washing away desquamated epithelial cells, foreign bodies and microbes [**2**].

The epithelia of the conjunctiva and the cornea act as mechanical barriers to infection [**3**]. Also, the conjunctiva has associated lymphoid tissue, termed CALT (conjunctiva associated lymphoid tissue), wherein immunity is initiated by exposure to exogenous antigens by the production of IgA antibodies [**4**].

## Risk Factors for Infective Keratitis

**Fig. 1:**
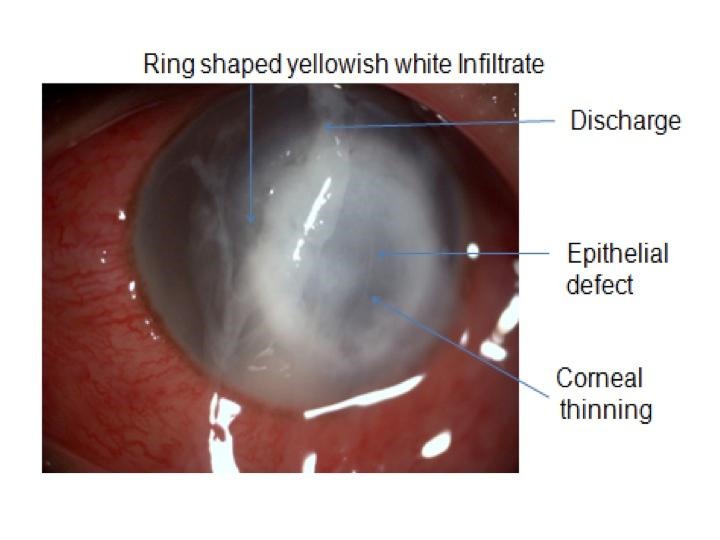
Slit Lamp photo of a patient presenting with infective keratitis

The cornea is at risk of infection when there is disruption of the defense mechanisms, which can be due to various local and systemic factors (**Table 1**) [**5**-**13**]. A systematic approach of infective keratitis in a patient (**Fig. 1**) not only helps establishing the correct diagnosis but also guides the further management of the patient.

## Clinical History and Examination

A detailed clinical history is necessary, which includes the duration of disease, any history of trauma, lacrimal sac infection, conjunctivitis, contact lens wear, or ocular surface disorder.

**Table 1:** Factors disrupting natural defense mechanism of eye

Local	Systemic
* Trauma to intact epithelium [**5**]	* Immunodeficient states [**12**]
* Contact lens wear [**6**,**7**]	▪ HIV-AIDS
* Eyelid-Entropion, Ectropion, Adnexal Infection	▪ Malignancy
* Neurotrophic disease	▪ Drug induced
* Bullous keratopathy [**8**]	* Connective tissue disorders like Rheumatoid arthritis (adversely affect corneal wound healing)
* Ocular Surface Disease [**9**,**10**]	* Diabetes
* Ocular Surface Disease [**9**,**10**]	* Measles, malnutrition and diarrhea [**13**]
* Topical medications - e.g. steroid	

It is also important to document if there were any similar episodes in the past signifying recurrence of disease and the current treatment of the patient, especially the use of steroids. Systemic history of diabetes, immune compromised states, immunosuppressive therapy, nutritional deficiency, and collagen vascular disease like Rheumatoid arthritis needs to be evaluated. Infective Keratitis is usually accompanied by redness, pain, watering, diminished vision, and intolerance to light. The patient needs to be evaluated for:

• Visual acuity, 

• Dimensions of the infiltrate and epithelial defect,

• Height of hypopyon,

• Anterior chamber reaction,

• Intraocular pressure, 

• Any ocular surface disorders,

• Ultrasonography for posterior segment to rule out endophthalmitis.

## Documentation

**Fig. 2:**
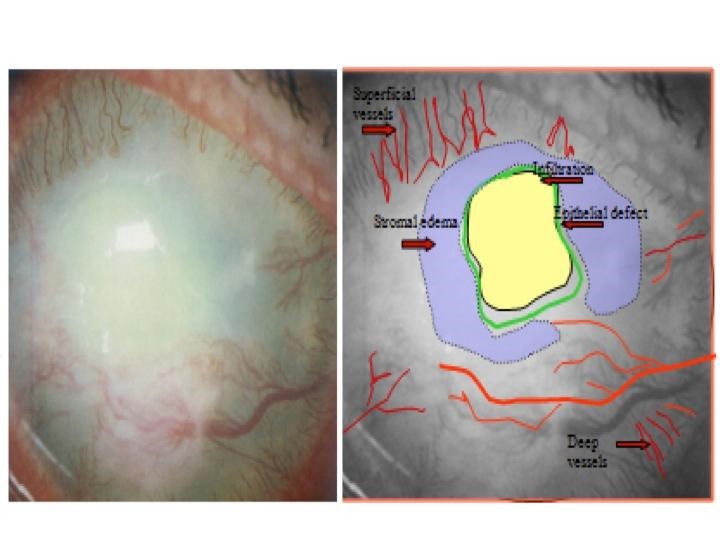
Clinical documentation with the help of color-coding

Documentation of keratitis is very important for management, follow up and assessment of therapy (**Fig. 2**). **Table 2** provides the color-coding for corneal diagrams [**14**]. The ulcer needs to be graded as mild, moderate or severe depending on its characteristics (**Table 3**) and its course from infiltration to healing needs to be documented (**Table 4**) [**15**].

**Table 2:** Color Coding for documentation

Epithelial Defect	Green
Stromal Edema	Blue
Epithelial Edema	Blue circle
Infiltrate	Yellow
Corneal Scar	Grey
Blood Vessels	Red (Complete lines)
Ghost Vessels	Red (Dotted lines)
Keratic Precipitates (KP)	Yellow
Pigments on endothelium	Brown

**Table 3:** Grading of corneal ulcer

	Mild	Moderate	Severe
Size	<2mm	2-5mm	>5mm
Depth	20%	20-50%	>50%
Infiltrate	Superficial	Mid-stromal	Deeper
Sclera	Not involved	Not involved	May be involved

**Table 4:** Stages of keratitis

Stage of progressive infiltration	Stage of active ulceration	Stage of regression	Stage of cicatrization
Stage of cicatrization	Necrosis and sloughing of epithelium, basement membrane, stroma	Induced by natural host immunity and treatment	Necrotic stroma replaced by scar tissue
Resultant destruction and necrosis of involved tissue	Margin and floor show grey infiltration and sloughing	Improvement in signs and symptoms Infiltrate decreases in size	Vessels regress completely or residual ghost vessels seen after healing

## Microbiological Workup

A good microbiological evaluation of infective keratitis is invaluable for a correct diagnosis, appropriate therapy and may improve the chances of a successful clinical outcome. Light microscopy and culture methods are commonly employed. Corneal scraping is performed at the slit-lamp under topical anesthesia. Proparacaine 0.5% eye drops should be preferred to lignocaine as it is less bactericidal [**16**,**17**]. A 26 or a 23-gauge needle, Bard Parker No. 15 blade or a Kimura spatula may be used. Any loose mucus or debris should be wiped away first. Carefully, the leading edge and the base of the ulcer are scraped. The material is transferred onto slides, at least two in number, for Gram stain and KOH. A circular mark with a pencil can be placed around the collected sample on the reverse side of the slide. In the same way, material is transferred to the culture media. If the patient is instilling several medications, it is preferable to stop antimicrobials for 24 hours and then scrape under close follow up.

## Microbiological staining

**Fig. 3:**
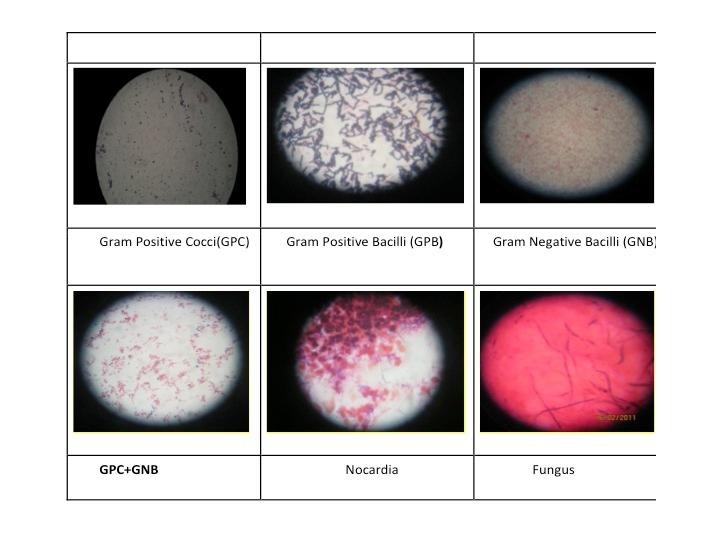
Various Gram stains used for diagnosis

Gram’s stain: Differentiates bacteria into two major groups, based on their ability to retain the dye crystal violet [**18**]. Can identify Acanthamoeba and Nocardia as well (**Fig. 3**).

**Fig. 4:**
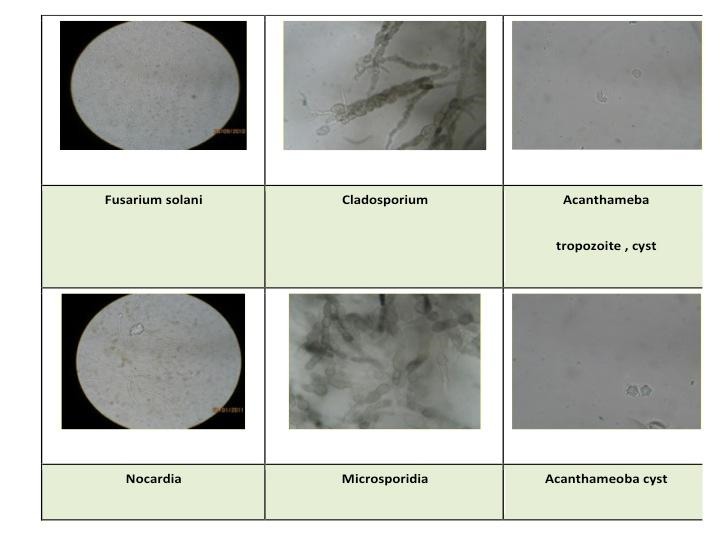
10% KOH wet mount used for diagnosis

**KOH wet mount** - Detecting fungus is very important so that indiscriminate use of antifungals is avoided as these medications are keratotoxic. Due to high sensitivity and specificity of KOH mount for fungus, this becomes the most important single test if there is lack of resources [**19**]. It is 92% sensitive and 96% specific for fungus [**19**]. It can also identify Acanthamoeba & Nocardia (**Fig. 4**).

The other specific stains not routinely performed are:

▪ **Giemsa stain** - It helps identify inclusion bodies in viral and chlamydia infections. It can also help differentiate bacteria and fungi, identify cysts and trophozoites of Acanthamoeba [**20**].

▪ **Ziehl-Neelsen stain** - for Mycobacteria, Actinomyces, and Nocardia [**21**].

**Fig. 5:**
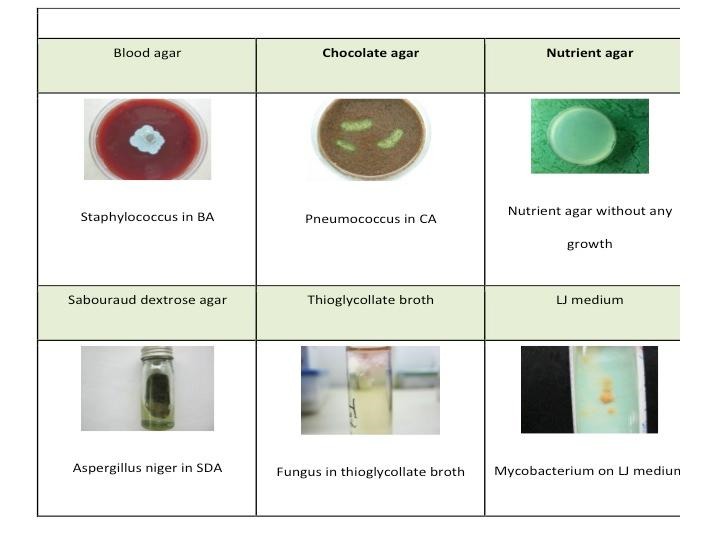
Various culture medias used in microbiology workup

**Culture Medias** - Bacterial and Fungal colonies have specific characteristics on culture that help in their identification (**Fig. 5**).

o Blood Agar - for isolation of aerobic bacteria at 35°C, and fungi and Nocardia at room temperature. 

o Chocolate Agar - for facultative organisms such as Haemophilus, Moraxella and Neisseria.

o Sabouraud Dextrose Agar - is a non-selective medium for opportunistic fungal pathogens.

o Non-nutrient Agar with E. Coli overlay for Acanthamoeba.

o Thioglycollate broth - facilitates the isolation of anaerobes.

o Lownstein Jensen (LJ) medium - for Mycobacteria.

## Clinical Features 

**Bacterial keratitis**

Bacterial keratitis is a common form of infective keratitis with incidence ranging from 50 to 60% [**22**] and Staphylococcus spp., Streptococcus spp. Pseudomonas being the frequent isolates. 

Patients usually complain of rapid onset of pain, photophobia, and decreased vision. An associated history of ocular trauma may be present. Biomicroscopic examination may reveal features such as ulceration of epithelium, suppurative stromal infiltration with surrounding edema, anterior chamber reaction with or without hypopyon and mucopurulent exudate.

Keratitis caused by Pseudomonas Aeruginosa, a Gram-negative organism is more fulminant and is associated with a worse visual prognosis than other bacterial pathogens [**23**]. Sudden onset and rapid progression of ocular pain is associated with epithelial defect and stromal infiltrate, which often assumes a ring configuration. Stromal necrosis and progressive thinning may be associated. Contact lenses are a common risk factor for keratitis.

**Fungal Keratitis**

Fungal keratitis has an incidence of 6 to 30%, with the common isolates being Aspergillus, Fusarium, Penicillium [**24**-**28**]. Fungal keratitis has certain special characteristics that may help differentiating it from bacterial Keratitis - feathery borders and indistinct edges [**29**], a fixed hypopyon [**30**], immune rings, endothelial plaques and a posterior corneal abscess [**31**].

**Parasitic Keratitis**

Acanthamoeba keratitis is a chronic, primarily contact lens-related infection caused by a free-living amoeba found ubiquitously in water and soil [**32**,**33**]. Acanthamoeba exists in two forms - trophozoites and cyst. Potential risk factors for Acanthamoeba keratitis are damage to the corneal epithelium, contact lenses, contaminated water, or solution and host susceptibility [**20**]. Clinical features of Acanthamoeba keratitis are severe ocular pain, epithelial irregularity and dendriform pattern, severe anterior and posterior scleritis, ring shaped stromal infiltrate, anterior uveitis, persistent or recurrent epithelial erosion, radial keratoneuritis, disciform keratitis [**20**]. Culturing the specimen on a confluent lawn of the E. coli plated on non-nutrient agar is the microbiological diagnostic technique. 

**Viral Necrotizing Keratitis**

It is a rare manifestation of HSV that results from direct viral invasion of the corneal stroma. The clinical findings are necrosis, ulceration, and dense infiltration of the stroma with an overlying epithelial defect. The combination of replicating virus and severe host inflammatory response leads to destructive intrastromal inflammation [**34**]. The clinical findings resemble those of infective keratitis secondary to microbial invasion. Therefore, necrotizing viral stromal keratitis must be considered a differential diagnosis. 

## Atypical organisms 

**Acid Fast Bacilli Keratitis**

Nocardia are aerobic, gram-positive, non-motile, and branched filamentous bacteria [**21**]. Trauma is the most common predisposing factor. The clinical picture of nocardia keratitis usually consists of superficial patchy infiltrates, which may be arranged in a wreath pattern [**21**]. Presence of gram-positive, branching, beaded filaments that stain with 1% acid-fast stain (using 1% sulfuric acid, modified Kinyoun’s method) is suggestive of nocardia infection. Nocardia grow on commonly used media as tiny, white, dry colonies [**35**]. Non-tuberculous or atypical mycobacteria are aerobic, non-spore forming, non-motile bacilli [**36**]. Infection can occur after corneal trauma, any anterior segment surgery, or use of contact lenses. Keratitis often manifests as a relatively indolent, recalcitrant stromal infection, which usually develops 2 to 3 weeks after trauma or surgery [**37**]. The disease may wax and wane over a period of several months. Pain is variable. The stromal infiltrates may show feathered edges, satellite lesions, crystalline keratopathy, or a ring pattern. A “cracked windshield” corneal infiltrate is virtually diagnostic [**36**]. The Fite Ferraco stain uses a gentler acid wash than the Ziehl-Nielsen technique, thus allowing the bacilli to better retain their stain [**37**]. Positive cultures on Lowenstein-Jensen agar medium constitute a definitive diagnosis. 

**Management**

Infective Keratitis patients can be managed as outpatients. The choice of specific antimicrobial agent hits the bull’s eye. Treatment is initiated based on smears, without waiting for the results of culture and sensitivity. Initial empirical therapy for bacterial keratitis involves frequent instillation of broad-spectrum antibiotic drops. Many prefer the combination mode of therapy, wherein; a cephalosporin is combined with an aminoglycoside. The cephalosporin covers the gram-positive cocci and some gram-negative rods, and the aminoglycoside, the gram-negative ones [**38**,**39**]. Commonly, 5% cephazolin is combined with 1.3% tobramycin. Monotherapy, using only one fluoroquinolone, is also effective. Ciprofloxacin 0.3%, Ofloxacin 0.3%, Gatifloxacin 0.3%, or Moxifloxacin 0.5% may be used [**40**,**41**]. However, monotherapy is usually reserved for keratitis, which is not severe or does not involve the visual axis.

Whatever the chosen strategy, monotherapy or combination therapy, drugs are started intensively. Initially, the loading dose is preferred followed by the hourly administration of topical antibiotic for the first 48 hours. These patients need a close follow up with corneal drawing depicting accurate measure of infiltrate, epithelial defect, and height of hypopyon.

Modification of drug therapy is required only if there is no response or worsening of the clinical signs on treatment. The drugs should not be changed if a favorable clinical response is seen, even if microbiological results show a resistant pathogen.

The use of topical corticosteroids as adjunctive therapy in the treatment of bacterial corneal ulcers has been debated extensively during the past few decades. The Steroids for Corneal Ulcers Trial (SCUT) conducted between 2006 and 2010 concluded that adjunctive topical corticosteroid use does not improve 3-month vision in patients with bacterial corneal ulcer [**42**].

Natamycin 5% ophthalmic suspension is the initial drug of choice for most cases of fungal keratitis. As in bacterial infections, dosing is started on an hourly basis, and then reduced as the ulcer started to resolve. For Candida infections, Amphotericin B 0.15% or Fluconazole 0.3% are effective. Voriconazole is a newer antifungal agent, an azole, with a broader spectrum of antifungal activity, and a lower minimum inhibitory concentration [**43**]. However, it is not recommended as monotherapy in filamentous keratitis. According to Mycotic Ulcer Treatment Trial (MUTT), Natamycin was associated with significantly better clinical and microbiological outcomes than voriconazole for smear-positive filamentous fungal keratitis [**44**].

Fungal keratitis is slower to respond than bacterial, taking several weeks [**45**]. Therefore, the duration of treatment is more prolonged. Oral antifungals are indicated in large or deep ulcers, scleral extension, or endophthalmitis. Fluconazole and Ketoconazole are the most widely used systemic antifungals. Two to three weekly liver function test assessments should be performed when a patient is kept on systemic antifungals.

Corneal epithelial debridement, every 24 to 48 hours, should be performed in fungal keratitis. It serves to debulk the cornea of necrotic debris and to enhance the penetration of topical antifungals. Therapeutic penetrating keratoplasty should be performed if medical therapy fails. Nearly a third of patients were found to fail medical therapy [**46**].

For Acanthamoeba keratitis, Chlorhexidine (0.02%), Polyhexanide biguanide (0.02%), Propamidine isethionate (0.1%) or Hexamidine (0.1%) are found to be effective [**20**]. In addition to topical medications, oral itraconazole (200 mg/ day) may be added.

For Necrotizing Viral Stromal Keratitis, therapeutic aqueous humor levels can be achieved with oral Acyclovir in a dose of 400 mg five times daily for 10 weeks and ointment Acyclovir 5 times a day for 2 weeks. Diluted steroids can be started after 2 weeks of treatment in tapering doses under careful observation [**34**]. Topical cycloplegics should be administered to relieve pain from ciliary spasm and prevent the formation of posterior synechiae. Homatropine, Atropine eye drops, or ointment may be given. A secondary glaucoma may accompany the anterior segment inflammation requiring topical 0.5% timolol twice daily or even systemic acetazolamide.

**Management of Keratitis caused by atypical organisms**

Nocardia Keratitis - Therapy with trimethoprim-sulfamethoxazole and amikacin is effective [**35**].

Atypical mycobacteria Keratitis - Topical fortified amikacin (14-100 mg/ ml) is the drug of choice. They may also show varying degrees of susceptibility to other drugs like fluoroquinolones, aminoglycosides, and tetracycline family [**37**].

**Assessment of Therapy**

There are specific biomicroscopic signs indicating the healing response in Keratitis. There is blunting of the perimeters of the infiltrate, reduction of the density of the suppuration with reduction in cellular infiltrate and edema in the surrounding stroma. Reduction in anterior chamber inflammation is noted with progressive re-epithelization and loss of the feathery perimeter of the stromal inflammation. 

If the keratitis is progressing then admission of the patient is advisable to ensure compliance. If resistance to the primary therapy is noted the microbiology results need to be reviewed and change to appropriate antimicrobials is advocated. Corneal biopsy is indicated and other possible causes must be investigated if the results are inconclusive.

## Surgical therapy

**Tissue adhesives**

Cyanoacrylate glue is indicated to manage perforations less than 3mm in size, and impending perforations and descemetocele. After thin glue application, bandage contact lens is placed over cornea.

**Therapeutic Keratoplasty**

**Fig. 6:**
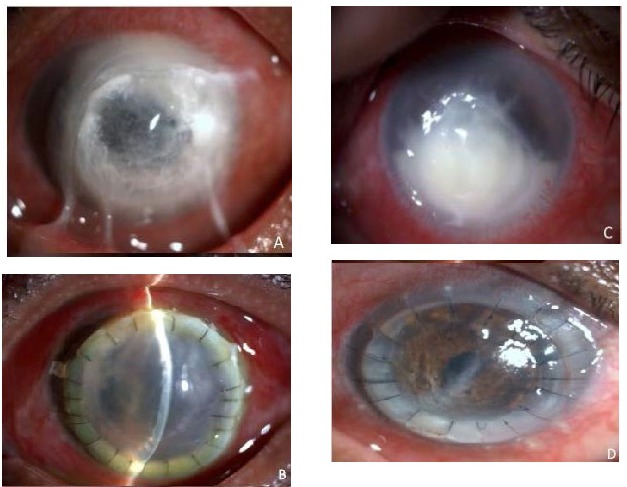
Bacterial keratitis leading to impending perforation treated with limbus-to-limbus keratoplasty (A) shows the pre operative picture (B) shows the postoperative picture (C) Non resolving fungal keratitis (D) therapeutic keratoplasty done for the same patient

Progressive ulceration, non-response to therapy or large areas of perforation requires a corneal graft (**Fig. 6 a-d**). The corneal button that is removed should be sent for microbiological evaluation. Also, the diameter of the trephination should be large enough to include 1mm of the surrounding normal cornea. Smaller areas of keratitis, requiring surgical treatment can be managed with patch grafts. 

## Conclusion

A meticulous clinical examination of a patient of infective keratitis goes a long way in arriving at the correct diagnosis. Appropriately, targeted antimicrobial therapy backed by microbiological investigations is the first step in the management of the condition. Keeping a bird’s eye view on progression, modifying therapy if required, and not delaying surgical intervention, if deemed necessary, is the key to a successful outcome.

**Acknowledgements**

None.

**Financial Disclosures**

Nil.

**Conflict of Interest**

None declared for any author.
